# Supplementary material for: Are carbon emissions trading and green financial instruments synergistic? -Comprehensive quantitative research based on content analysis
Source: PLoS One. 2024 Mar 7;19(3):e0298601. doi: 10.1371/journal.pone.0298601 (PMC10919601; doi:10.1371/journal.pone.0298601)
Supplement: S1 Data — (ZIP) [file pone.0298601.s001.zip › Data/地级市Z_F工作报告.zip!/░▓╗╒/╗┤─╧╩╨/└┤╫╘╣1⁄2╓┌║┼í╛┬φ┐╦╩2╛▌═°í┐_2019.docx]

各位代表：

现在，我代表市人民政府，向大会报告政府工作，请予审议，并请市政协委员和其他列席人员提出意见。

**一、2018年工作回顾**

刚刚过去的2018年，是全面贯彻党的十九大精神的开局之年。在省委、省政府及市委的坚强领导下，我们坚持以习近平新时代中国特色社会主义思想为指导，坚持稳中求进工作总基调，按照高质量发展要求，全面落实“六稳”部署，紧扣“1235”发展思路，积极应对经济下行压力，深入开展“五个年”活动，坚定信心，迎难而上，扎实工作，保持了经济平稳健康发展和社会大局稳定，朝着实现全面建成小康社会的目标迈出了新的步伐。预计，全市地区生产总值1130亿元，增长4%；财政收入173.9亿元，增长7.2%；社会消费品零售总额614亿元，增长11.5%；固定资产投资增长2.4%；城乡常住居民人均可支配收入分别达32898元和12859元，分别增长8.2%、8.6%。

——产业结构优化升级。预计，三次产业结构为10.6:47.4:42，服务业增加值比重提高0.7个百分点。非煤电规上工业增加值增长8%，高新技术产业增加值、战略性新兴产业产值分别增长23%、17.5%。省级以上园区规上工业增加值增速高于全市6.5个百分点。单位地区生产总值能耗下降6%左右。

——项目建设扎实推进。中安煤化一体化项目一期建成中交，引江济淮淮南段、网云小镇等开工建设，商合杭高铁淮南段及站点、恒大阳光半岛、皖江物流综合码头、光电产业园等加快推进，淮滁高速、盖天力医药产业园、中储粮淮南直属库等建成投用，总投资27.8亿元的谢家集重点采煤沉陷区综合治理项目获国家批准。

——发展活力加速释放。长三角区域一体化发展、淮河生态经济带上升为国家战略，为淮南带来前所未有的发展机遇和战略红利。高新区升格为国家高新技术产业开发区。合淮产业走廊规划获省政府审议通过。“中国成语典故之城”申创成功。各类市场主体增长30.7%。新增“四上”企业134家。授权发明专利410件。实现直接融资255亿元，金融机构贷款增量创历史新高。

——质量效益持续提升。规上工业企业利润增长10%。PM2.5、PM10平均浓度分别下降10.1%、18.1%，优良天数比例提高6.6个百分点。居民消费价格指数温和上涨。城镇新增就业6.1万人，农村劳动力转移就业4.88万人。43310人脱贫，44个贫困村出列，脱贫攻坚取得决定性进展。

一年来，主要抓了以下七个方面工作。

一是全力打好三大攻坚战，坚决扛起政治责任。切实提高政治站位，防风险补短板强弱项取得重要进展。打好防范化解重大风险攻坚战。规范政府举债融资，全面完成存量政府债务置换。开展“四清四实”专项整治，严厉打击非法集资等行为。引导金融机构加大对实体经济支持力度，新增省区域性股权交易市场挂牌企业70家，发放新型政银担贷款、“税融通”贷款、续贷过桥资金85亿元。打好精准脱贫攻坚战。扎实开展脱贫攻坚作风建设年活动，实施打赢脱贫攻坚战三年行动，开展四级书记遍访贫困对象推进乡村振兴行动。出台防范返贫确保稳定脱贫等政策文件，各级财政安排扶贫资金10.2亿元。“351”“180”健康脱贫政策惠及贫困患者30万人次。加强行蓄洪区脱贫攻坚，编制基础设施建设方案。举全市之力推进革命老区、国家级贫困县寿县脱贫摘帽。打好污染防治攻坚战。强力推进环保督察反馈问题整改，严格落实河长制、湖长制、林长制，坚决打好蓝天、碧水、净土保卫战。全市环保项目总投资约105.4亿元、完成37.9亿元，其中政府性投资约55.6亿元。备受关注的一污厂二期扩建工程、一水厂取水口上移工程竣工投用，95个入河排污口完成整治，城市黑臭水体治理初见成效。全面完成7个县级以上集中式饮用水水源地整治。新改建各类管网137.1公里，新增污水处理能力8.5万吨/日。全国水生态文明城市试点通过验收。出台餐厨废弃物管理办法。启动中心城区污水工程专业规划编制工作。完成成片造林1.54万亩。46平方公里西部采煤沉陷区生态治理概念性规划编制完成。世行贷款采煤沉陷区综合治理项目加快建设。

二是全力推动产业升级，新旧动能逐步转换。落实省委打好“四张资源牌”要求，推动转型提速高质量发展。提升煤电产业。全年生产原煤7100万吨，发电700亿千瓦时。煤制乙二醇、煤制气、潘集电厂等项目前期扎实推进，潘集选煤厂、三峡新能源光伏等项目建成投运。国家第三批增量配电业务改革试点项目获批。中国煤电固废生态治理与循环经济发展大会在淮成功召开。壮大非煤产业。出台支持制造强市建设、降低企业成本等政策文件，进一步调动市场主体的积极性。建成安徽大数据交易平台，省级大数据产业基地荣获长三角大数据应用（智慧城市）服务贡献奖。全年新增规上工业企业86家、省级以上绿色工厂8家。高新区在2017年度全省开发区综合考评中位列第17位。毛集实验区跻身2017年度省制造业发展增速10快区。拓展现代服务业。新城吾悦、万达广场等商贸综合体开业。恒大空港特色小镇等项目正式签约，安徽楚文化博物馆、毛集花田花海等项目开工建设，寿县古城游客集散中心等项目建成。新增限上商贸单位91家。旅游人数、旅游总收入分别增长16.5%、18.5%。文化事业加快发展，文化产业增加值17.4亿元。花鼓灯舞蹈等一批作品获国家奖项，寿县跻身中国地名文化遗产“千年古县”行列。

三是全力推进项目建设，发展基础不断夯实。把项目作为稳投资的“压舱石”、促转型的“牛鼻子”，紧抓不放、久久为功。紧抓项目建设提速。亿元以上省重点项目新开工115个、续建157个、竣工42个，完成投资444亿元，完成年度计划的120%。紧抓招商引资突破。到位亿元以上内资545亿元，引进亿元以上省外项目资金460亿元，新开工5000万元以上招商引资工业项目102个。恒大高科农业生产基地、中国中车淮南电机研发和生产基地等一批产业项目签约。紧抓遗留问题攻克。坚持“依法合规、尊重历史、实现双赢”原则，奥体中心、迎宾馆等17个政府性投资项目问题处置实现突破，金融世家、淮河新城五期等11个房地产项目问题得到化解，安粮淮安府等7宗土地通过司法、行政等途径盘活，其他历史遗留问题也在积极推进。紧抓营商环境优化。建立“四最”营商环境评价体系，调整涉企收费清单，降低城镇土地使用税等级税额标准。建立“四送一服”常态化推进机制，为企业解决各类问题2247件，在全省“四送一服”双千工程集中活动专项评估中获得第四名。

四是全力推进改革开放创新，发展红利稳步释放。深化供给侧结构性改革，扎实推进121项改革任务，隆重开展改革开放40周年庆祝活动。抓好“去降补”工作。关闭退出1对煤矿、600万吨产能，妥善分流安置职工7546人。国有企业“三供一业”分离移交工作顺利完成。平稳有序实施盐业体制改革、5家国有农场剥离办社会职能改革。房地产市场平稳健康发展。落实结构性减税政策，全年减税降费24.2亿元。推进“放管服”改革。落实“六个一”要求，企业开办时限压缩至3个工作日，不动产证办理时限压缩至4个工作日。推进政务服务“一网、一门、一次”改革，90%以上进驻事项实现“最多跑一次”。“减证便民”申请材料精减56.9%，“证照分离”改革全面推开。扩大开放合作。进出口总额增速居全省前列，实际到位外商直接投资增长9%。与合肥共建的寿蜀产业园有序发展，主要经济指标均保持25%以上增长。与央企、国企、知名民企合作稳步推进。与塞尔维亚武贝市建立友好城市关系。成功举办淮南首届“一带一路”境外商品展。助力民营经济发展。认真落实习近平总书记在民营企业座谈会上的重要讲话精神，出台大力促进民营经济发展的若干意见，全年新增民营企业9412家、个体工商户25345家。实施创新驱动发展战略。出台支持科技创新若干政策。新增高技能人才5016人。主导、参与制定国家、行业标准8项，建成全省唯一市级承建的国家级最高计量标准。在全省率先成立市级缺陷产品召回中心。新增重大新兴产业专项1个、省级博士后工作站3家、省级青年创业园1家、省级科技企业孵化器2家、省级众创空间6家。新增高新技术企业25家、中国驰名商标1件。2个项目入选省院地合作重大项目库。中科院淮南大气环境科技产业园加快建设，淮南新能源研究中心研发区一期建成投用。安理大省部共建国家重点实验室获批建设。“煤炭安全智能精准开采”中国工程科技论坛暨“协同创新组织”年会在淮举办，24位中国工程院院士集聚淮水之滨、共谋创新大业。

五是全力促进区域协调，城乡发展加速融合。引导“住建规划下乡、水利林业进城”，让城市与乡村、自然与人文各美其美、美美与共。主攻山南建设。围绕聚项目、聚人气，完成投资145亿元。编制山南新区总体城市设计及六大导则。4所小学、4家民办医疗机构、2个菜市场、1座加油加气站建成投用，互联网经济产业园一期、国家煤化工质检中心二期等项目顺利完成，碧桂园、龙成奥韵星级酒店等36个社会投资项目加快建设。提升老城品质。完成淮凤路、卧龙山路及安成铺周边道路改造、11条城市道路大中修，建成十涧湖、大涧沟城市大型排涝泵站。推动5个公交枢纽站场建设，新增公交车130辆。改造棚户区2436套、城中村2880套、老旧小区43个。整治小街小巷100条，升级改造老龙眼等菜市场23个，建成舜耕山湿地公园、和平广场公园、13个街头游园，这些群众“身边事”的实施赢得了众多点赞。推动乡村振兴。落实藏粮于地、藏粮于技战略，全年粮食产量320.2万吨。实施农业产业化五大工程，新增农业产业化龙头企业31家，规上农产品加工产值增长10%。“潘集酥瓜”“凤台平菇”获批中国地理标志证明商标。完成“两区”划定、农村电商全覆盖巩固提升行动。31个乡镇政府驻地建成区和48个省级中心村完成整治建设，乡镇政府驻地、乡村学校旱厕和5万户自然村农厕完成改造。完成3691户农村危房改造。开工沉陷区安置房3200套、搬迁入住3600户。完成农村道路畅通工程435.1公里。建成西淝河、高塘湖大型泵站。解决12.18万农村人口饮水安全问题。毛集实验区入选全国农村集体产权制度改革试点。出台县域经济振兴支持政策，推动县域经济创新发展。创建文明城市。建立路长制，实施八大提升行动。推进志愿者服务常态化，发布推广《淮南文明18条》。7人当选中国好人，1人荣获全国五一劳动奖章。群众对创城工作的知晓率、支持率和满意率不断提高，城市形象、市民素质进一步提升。

六是全力推进共建共享，人民福祉持续增进。全市民生支出197.5亿元，占财政支出的80.6%。33项民生工程投入47.8亿元，占年度计划的109.9%。五项社会保险参保411.9万人次，城市和农村最低生活保障标准、特困供养人员财政补助标准、孤儿保障标准、困难残疾人生活补贴标准、重度残疾人护理补贴标准分别提高8.6%、70%、34%、10%、5%、16.6%。坚持教育优先发展，在全省率先启动县域义务教育优质均衡发展创建，学生资助工作获教育部表彰。实施积极的就业政策，发放创业担保贷款3.16亿元、企业稳岗补贴7774万元，城镇调查失业率控制在5%以内。推进健康淮南建设，智慧医疗项目基本建成，公立医院改革扎实推进，社会办医规模稳居全省首位。第七次荣获全国无偿献血先进城市称号。成功创建省级食品药品安全城市，首届安徽省食品安全进校园活动在淮举办。成功组织淮南国际马拉松等赛事，在省第十四届运动会上取得总分第六的好成绩。淮南应急广播正式开播。圆满完成村（居）民委员会换届工作。深入开展“深重促”专项行动，化解信访突出问题127件。深化平安淮南建设，重拳扫黑除恶，安全生产形势总体平稳，社会大局保持和谐稳定。信访工作连续两年被评为全省目标考核优秀单位。推动军民融合深度发展，成立退役军人服务管理中心。民族宗教、外事侨务、对台、工会、共青团、妇女儿童、老龄、残疾人、气象、红十字、档案、地方志、人防、地震、应急管理、机关事务管理、援藏援疆等事业取得新进展。

七是全力加强政府自身建设，行政效能不断提升。始终坚持党的全面领导，建立市政府党组向市委请示报告等制度，着力增强八种本领，切实优化工作作风。扎实开展“全面学合肥，对标提效能”活动。把学习活动延伸到科室长、办事员、基层单位和服务窗口，对标学习合肥先进经验、创新做法、制度流程，建章立制，提升行政效能。政务服务满意率达到96.98%。市长热线荣获全国12345政府热线“接通新星奖”、人民网网民留言办理先进单位。严格依法行政。完成全市“七五”普法中期督查。重大事项合法性审查、政府法律顾问实现全覆盖。提请市人大常委会审议地方性法规草案2件。办理人大代表议案建议368件、政协委员提案335件。田家庵区荣获全国法治县（市、区）创建活动先进单位。全面从严治政。深入开展“讲严立”专题警示教育，持续深化“三查三问”。树牢过紧日子思想，主动压减调整市本级预算支出1.07亿元，全市“三公”经费持续下降。坚持不懈推进党风廉政建设和反腐败斗争，加强审计监督，锲而不舍纠“四风”、转作风。

各位代表！过去一年，我们踏上新时代高质量发展的新征程，肩负着开创性使命；我们经历了多重困难风险的挑战，付出了很多艰辛。面对经济下行的较大压力、产业结构的突出矛盾、防风险补短板的艰巨任务，经济能够稳得住很不容易，出现诸多向好变化更为难得。这是习近平新时代中国特色社会主义思想科学指引的结果，是省委、省政府及市委坚强领导的结果，是全市广大干部群众团结奋斗的结果。在此，我谨代表市人民政府，向辛勤工作在各条战线的干部群众，向给予政府工作大力支持的人大代表、政协委员，向各民主党派、工商联、无党派人士、人民团体和社会各界人士，向驻淮部队、武警官兵、公安干警和消防救援人员，向所有支持淮南改革发展的同志们、朋友们，表示衷心的感谢！

同时，我们必须清醒地看到，经济运行稳中有变、变中有忧，发展不平衡不充分的问题还比较突出。主要体现在：一是经济下行压力较大，受外部环境复杂严峻、内部调整优化升级等多重因素影响，地区生产总值、固定资产投资等指标没有完成预期目标，特别是项目支撑不足，固定资产投资增速回落需要引起高度重视。二是新旧动能接续转换任务艰巨，创新发展能力不强，产业结构依然不优，非煤产业、民营经济发展滞后，农业抗风险能力需要加强。特别是实体经济发展面临不少困难，营商环境需要进一步改善。三是县区发展不充分，园区经济总量不大，开放型经济发展水平不高，招商引资不足，凤台县、田家庵区、谢家集区、八公山区、潘集区、市经开区、市高新区、市煤化工园区未完成规上工业企业培育年度任务。四是生态环境保护任重道远，污染防治、采煤沉陷区综合治理等仍需加大力气。五是民生领域还有不少短板，教育、医疗等公共服务优质化均衡化水平有待提升。六是一些干部专业素养需要提升，政策制定和落实能力有待提高，少数干部不担当不作为、形式主义、官僚主义不同程度存在。这些问题是前进中的问题，既有短期的也有长期的，既有周期性的也有结构性的。对此，我们要增强忧患意识，抓住主要矛盾，有针对性地加以解决，决不辜负全市人民的重托！

**二、2019年重点工作**

今年是新中国成立70周年，是全面建成小康社会关键之年。在庆祝改革开放40周年大会上，习近平总书记发出了“将改革开放进行到底”的动员令，对淮南而言，推动改革开放再出发，更具历史意义、现实意义和长远意义。“走千走万，不如淮河两岸”。淮南承东启西、连南接北、左右逢源，是联结我国几大重要经济板块的重要节点；淮南历史悠久、文化璀璨、山清水秀，有着“楚风汉韵、能源之都”的独特魅力。中央“六稳”等重大政策红利在持续释放，长江经济带、长三角区域一体化发展、淮河生态经济带、合肥都市圈等重大战略平台在集中叠加，煤电、数据、科教、山水生态文化等重大资源在协同转化，航空、高铁、高速公路、水运等交通优势在拓展提升，中安煤化一体化、引江济淮、商合杭高铁等重大项目在加快建设，淮南发展仍处于并将长期处于大有可为的重要战略机遇期。我们必须从长期大势认识当前形势，认清淮南长期向好的发展前景，坚定必胜信心，抓住重大机遇，担当历史使命，振兴淮南经济。

今年工作的总体要求是：坚持以习近平新时代中国特色社会主义思想为指导，全面贯彻党的十九大和十九届二中、三中全会精神，深入贯彻落实习近平总书记视察安徽重要讲话精神，全面贯彻中央经济工作会议及省委、市委经济工作会议精神，统筹推进“五位一体”总体布局，协调推进“四个全面”战略布局，牢牢把握并全面用好重要战略机遇期，坚持稳中求进工作总基调，坚持新发展理念，坚持推动高质量发展，坚持以供给侧结构性改革为主线，坚持深化市场化改革、扩大高水平开放，按照“1235”发展思路，着力推进五大发展行动计划，继续打好三大攻坚战，着力激发微观主体活力，统筹推进稳增长、促改革、调结构、惠民生、防风险，进一步做好“六稳”工作，提振市场信心，增强人民群众获得感、幸福感、安全感，保持经济持续健康发展和社会大局稳定，为全面建成小康社会收官打下决定性基础，加快建设现代化五大发展美好淮南，以优异成绩庆祝中华人民共和国成立70周年！

今年发展的主要预期目标是：地区生产总值增长6-6.5%，财政收入增长7%，固定资产投资增长6%，社会消费品零售总额增长9%，城乡常住居民人均可支配收入分别增长7%、8%，城镇调查失业率5.5%左右，节能减排完成省下达的目标任务。

各位代表！发展是解决淮南一切问题的基础和关键。抓发展，是政府的本职，也是天职。做好今年工作，需要把握好以下原则。

一是坚持新发展理念科学指引不动摇。把“稳”放在首要位置，把“进”作为关键支撑，以更有力的“进”，把“稳”的基础打得更牢固。打好“四张资源牌”，做好“三篇文章”，处理好危和机的关系、绿水青山和金山银山的关系、存量和增量的关系、点和面的关系、当前和长远的关系、补短板和防风险的关系，构建“一主一副四组团”城市空间格局，推动转型提速高质量发展。

二是坚持以人民为中心发展思想不动摇。践行全心全意为人民服务的根本宗旨，及时回应社会关切，把群众的小事当作我们的大事。坚持尽力而为、量力而行，抓重点、补短板、强弱项，一件事情接着一件事情办，一年接着一年干，让全市人民生活一年更比一年好。

三是坚持弘扬改革开放伟大精神不动摇。落实“巩固、增强、提升、畅通”八字方针，持续深化供给侧结构性改革。加快思想大解放、环境大改善、能力大提升，创优“四最”营商环境。积极融入国家战略、省级平台，努力形成全方位、多层次、宽领域的全面开放新格局。

四是坚持“项目即发展”行动自觉不动摇。坚守“项目为王、项目为上”工作导向，一手抓“顶天立地”的大项目，一手抓“千家万户”的小项目，以项目成效检验干部实绩。持续推动“开发区+招商+规上工业企业培育”三位一体突破，加快投资结构、产业结构、税收结构优化升级。

五是坚持党的领导根本保证不动摇。坚持党的基本理论、基本路线、基本方略，始终把发展作为第一要务，始终以经济建设为中心，激励干部担当作为，营造良好发展环境。落实全面从严治党要求，自觉在市委领导下开展工作，确保政令畅通、令行禁止，努力营造积极向上、风清气正、干事创业的良好政治生态。

各位代表！推动转型提速高质量发展是一项长期、曲折、艰苦的系统性工程和历史性任务，是一场接力跑，我们要一棒接着一棒跑，一棒更比一棒好。今年重点做好八个方面的工作。

（一）深化改革创新，增强转型提速高质量发展的引领性。推动改革创新走深走实，加快质量变革、效率变革、动力变革。聚力供给侧结构性改革。做好去产能和“三供一业”分离移交后续工作，多措并举盘活退出矿井存量资源。稳步推进企业优胜劣汰，加快处置“僵尸企业”。坚持“房子是用来住的，不是用来炒的”定位，完善住房市场体系和住房保障体系，促进房地产市场平稳健康发展。用2年时间基本解决房地产领域群众入住、办证等历史遗留问题。推进增量配电业务改革试点。创新要素配置机制，引导金融、土地、能源、环境容量等要素向优质高效领域流动。落实降低社会保险费率等政策举措，清理、精简行政审批事项和涉企收费，让实体经济轻装前行。抓住国家基础设施补短板政策机遇，争取更多政策红利。聚焦主要矛盾，加强政策协同，努力实现最优政策组合和最大整体效果。聚力重大风险防范化解。坚持结构性去杠杆的基本思路，做到“坚定、可控、有序、适度”。落实政府性债务管理制度，推进建发集团、产发集团市场化转型，妥善化解政府隐性债务风险，争取一批适用政府专项债券的大项目、好项目。健全地方金融监管协调机制，推进地方金融机构不良资产压降行动，持续整治互联网金融，坚决遏制非法集资。优化金融服务监管和激励考核，保持信贷稳定增长，扩大直接融资规模，加大金融机构引进力度，提高金融体系服务实体经济能力。支持企业赴上交所科创板上市。推动多层次、普惠性农业保险体系建设。聚力重点领域改革。按时完成市、县区政府机构设置和职能配置。深化“放管服”改革，建好全市政务服务“一张网”，推行一窗受理综合服务，实现线上“一网通办”、线下“只进一扇门”。深化商事制度改革，全面推行“双随机、一公开”跨部门联合抽查监管。做好第四次经济普查、第三次土地调查。持续深化国资国企、财税体制、重点领域价格、医疗卫生等领域改革。聚力创新驱动发展。大力实施“十三五”全民科学素质行动计划纲要。创建国家新型智慧城市，积极融入合肥滨湖科学城建设。建立与大院大所常态化对接机制，支持安理大省部共建国家重点实验室和科技创新园区建设，加快光电产业园项目进程，提升科技成果就地转化水平，推动科教资源向发展竞争力转变。全面释放“双创”活力，推进“1+3+8”科技企业孵化器体系建设，申请发明专利1500件，新增省级众创空间2家、高新技术企业20家。大力弘扬劳模精神和工匠精神，实施新时代“江淮英才计划”，抓好科研项目评价、科研人员激励等政策落实，新增高技能人才5000人。深入推进质量提升行动，建设质量强市。以资源理念看待采煤沉陷区、棚户区、城中村和煤电固废物，积极推进探索性发展、创新性发展、引领性发展。

（二）聚焦战略平台，增强转型提速高质量发展的支撑性。扩大对外开放，统筹“五外”联动，抢抓战略机遇，开启融入发展新篇章。全面实施准入前国民待遇加负面清单管理制度，进出口总额增长10%，外商直接投资增长7%。全面对接长江经济带战略平台。深度融入长三角区域一体化发展战略，落实长三角地区一体化发展三年行动计划，复制推广上海自贸区改革试点经验，在空间规划、科技创新、产业布局、市场要素、生态环保、基础设施和公共服务等领域开展全方位合作，打造承接产业转移示范基地。坚持融入工作项目化，做好商合杭高铁淮南段如期通车相关工程建设，谋划一批事关长远的重大项目，打造长三角能源原材料和数据资源的重要保障基地。全面对接淮河生态经济带战略平台。统筹考虑水资源、水生态、水安全、水景观建设，制定淮河生态经济带发展规划淮南行动计划，谋划百亿对接项目库。科学规划淮河岸线资源，优化沿淮产业和城镇布局。主动对接国家政策，在重大工程、资金投入、体制创新等方面做好政策储备。扎实推进引江济淮淮南段、正峡段及峡涡段行洪区调整和建设等工程，加快皖江物流综合码头、中安联合茨淮新河码头等建设，谋划北沿淮高速项目，为淮河生态经济带建设贡献淮南力量。全面对接合肥都市圈战略平台。落实省委“把合肥都市圈建设成为世界级城市群的区域中心”的部署，主动融入、精准接轨，谋划百亿融合项目库，在更高层次上推动规划、交通、产业、环保、社会事业等协同发展。深入推进合淮同城化，实施合淮产业走廊规划，探索与合肥市经开区合作推进空港示范区联动发展，支持寿蜀产业园建设。推动合肥海关淮南办事处升格为淮南海关。开工建设恒大空港特色小镇等项目。大力发展数字经济，实施大数据产业发展三年行动计划，推进安徽大数据技术标准创新基地、安徽大数据交易中心建设，打造大数据生产、存储和应用基地。以省级大数据产业集聚发展基地为依托，进一步发挥钱学森智库淮南中心作用，加快光纤传感、智能终端、高端软件等发展，合作共创长三角国家大数据综合试验区。建成风云四号气象卫星省级直收站，谋划建设大数据加工场等项目。进一步扩大4G网络覆盖面，统筹5G网络规划建设。拓展融合型数字经济领域，积极培育共享经济、平台经济。大力发展“数字+”社会服务，推进电子商务、智慧交通、智慧旅游、智慧物流等建设。

（三）统筹项目投资招商，增强转型提速高质量发展的稳定性。建立“开发区+招商+规上工业企业培育”三位一体领导机制、推进机制，持续发挥投资关键作用。继续实施规上工业企业培育三年行动计划，净增规上工业企业100家。以项目为抓手。继续开展项目建设提速年活动，注重在项目审批、要素供给、项目调度上提速。坚持层级调度、一线调度，市级领导联系包保百个重点项目。谋深谋细项目前期，动态储备亿元以上项目600个以上。开工建设合霍阜高速淮南段、中至信农副产品交易中心等100个亿元以上重点项目，加快建设西部垃圾发电厂改扩建工程等155个亿元以上重点项目，建成淮上淮河大桥、奥体中心等40个亿元以上重点项目。继续开展历史遗留问题攻克年活动，依法稳妥处理项目建设中规划、土地等历史遗留问题。以招商为路径。继续开展招商引资突破年活动。加强招商队伍建设，坚持产业招商，开展组团招商，加强驻点招商，突出园区招商，善于利用高能级开放平台招商。完善重大项目考察和重要客商来访接待机制、招商利益共享机制、招商项目后评价机制，强化合同履约率、资金到位率、项目开工率考核，确保引进亿元以上省外项目资金平稳增长。以开发区为平台。坚持“亩产论英雄”“环境论英雄”，实施开发区高质量发展行动，在打造百亿园区上实现突破。创新开发区管理体制，试点“管委会+公司”模式，探索“一园多区”“跨区托管”方式。推动开发区规划修编，明确主导产业特别是首位产业，加速特色化、差异化发展。强化要素支撑，研究差别化土地供应政策，引导金融机构在开发区设立分支机构，探索建立“一枚公章管审批”等机制。最大限度下放管理权限，加快建立全程代办制，推广容缺后补、绿色通道、首席服务官等便利化措施。支持开发区在政策允许和权限范围内，制定招商引资优惠政策。理顺开发区与代管乡镇、街道的关系，真正让开发区轻装上阵。以县区为阵地。坚持扬长避短、扬长补短，进一步明晰各区域功能定位、产业选择和主攻方向，加快县区经济振兴。建立县区政府政策研究和谋划项目工作联席会议制度，县区政府明确一名同志主抓经济、项目工作。全面落实支持县域科技创新政策，支持两个县发挥比较优势，推进县域经济高质量发展；支持市辖区加快传统产业转型升级，发展新技术、新产业、新业态、新模式“四新经济”，形成多点支撑、竞相发展格局。以民营经济为生力军。建立健全促进民营经济发展的领导机制、工作机制和企业投诉维权机制，建立市领导帮扶百家重点企业机制，帮助企业解决转型、融资、市场开拓等难题。强化政银企对接，整合设立1亿元以上市中小企业（民营经济）发展专项资金，设立1000万元进出口企业助保金风险资金池，新增新型政银担业务25亿元。推动政策性融资担保体系建设，引导金融机构扩大对民营企业、小微企业信贷投放。开展清理拖欠民营企业、中小企业账款专项行动。实施民营企业培育提升工程，新增民营企业5000家、个体工商户1.3万家，培育省级“专精特新”中小企业10家以上。普遍落实“非禁即入”原则，鼓励民营资本参与国有企业混合所有制改革，鼓励民营企业公开公平公正参与市场竞争。弘扬企业家精神，支持企业家“二次创业”，评选表彰一批优秀民营企业和优秀民营企业家。以环境为保障。继续开展营商环境优化年活动。健全市县（区）营商环境评价分析系统，做好商务成本、时间成本、制度交易成本“三个减法”和公共服务、环境保护、文明文化“三个加法”。落实好减税降费政策，推进工程项目行政审批制度改革，加大公平竞争审查力度。构建亲清新型政商关系，常态化开展“四送一服”双百工程，健全企业家参与涉企政策制定机制，当好“店小二”，服务企业家。

（四）做好“三篇文章”，增强转型提速高质量发展的持续性。建立“一个产业、一套班子、一个平台、一个规划、一套政策、一抓到底”的“六个一”推进机制，力争用3-5年时间打造煤化工、大数据、高端金属制造、陕汽及其配套、煤矿开发伴生资源综合利用等多个百亿产业。做好煤电资源文章。做精做优煤电化产业链，推动煤电化一体化发展，打造绿色能源、绿色化工。建立健全市矿协调机制，支持淮南矿业整体改制上市、中煤新集转型发展，保障中安煤化一体化项目一期建成投产，加快推进煤制乙二醇、煤制气、潘集电厂等项目前期。按照“气衍生、灰衍生、热衍生”的思路，综合利用火电余热，建设潘集、凤台煤系固废综合利用产业园。做好非煤产业文章。推动制造业高质量发展，加快重大新兴产业基地、工程、专项建设，推进先进制造业与现代服务业深度融合，向产业链和价值链高端迈进。实施年产20万吨新兴医药原料、中高端数控设备制造等60个亿元以上技改项目，推进谢家集宏泰钢铁二期、大通高端制造产业园等项目建设，完成工业投资260亿元、技改投资170亿元。深入实施现代服务业集聚发展工程，大力推进高新区大数据产业、寿县“互联网+”等集聚区发展，服务业增加值增长6%以上。做好山水生态文化文章。强化全域理念、规划融合和区域合作，积极争创3个国家5A级旅游景区，完成八公山风景名胜区控制性详细规划编制，支持寿县、毛集创建国家全域旅游示范区，打造全国重要的文化旅游目的地。丰富中国成语典故之城内涵，加快安徽楚文化博物馆、寿春城考古遗址公园等建设。支持寿县申报中国民间文化艺术之乡，支持田家庵区创建全国公共文化示范区，支持大通区红色文化旅游基地建设。文化产业增加值增长10%，旅游人数、旅游总收入分别增长17%、19%。启动谢家集采煤沉陷区综合治理试点项目建设，加快推进潘集矿区采煤沉陷区综合治理项目前期，支持八公山区申报国家重点采煤沉陷区综合治理试点区，稳步推进沉陷区“华丽转身”。编制养老服务设施布局规划，推动医疗卫生、健康养老和山水生态融合发展，建设综合康养产业区。

（五）大力实施乡村振兴战略，增强转型提速高质量发展的协调性。聚焦乡村振兴总目标、总方针、总要求，完成乡村振兴战略规划编制，推进城乡功能、城乡环境、城乡改革一体提升、一体改善和一体突破，加快城乡一体化进程。深入推进脱贫攻坚。继续开展打赢脱贫攻坚战三年行动，持续实施四级书记遍访贫困对象推进乡村振兴行动，深入推进“十大工程”，大力推广“四送四通”扶贫直通车模式，一鼓作气完成19757人脱贫、11个贫困村出列。统筹推进贫困村与非贫困村脱贫攻坚，聚焦革命老区和行蓄洪区，健全稳定脱贫长效机制，织密扎牢“两不愁三保障”网底。研究解决收入水平略高于建档立卡贫困户的群体缺乏政策支持等新问题。全力以赴支持寿县做好贫困县摘帽退出评估验收工作。加快推进行蓄洪区安全建设。把扶贫与扶智扶志深度融合，激发脱贫内生动力。加大考核评估、巡视巡察中发现问题整改力度，持续推进扶贫领域作风建设。积极发展现代农业。坚持农业农村优先发展，出台支持现代农业发展的实施意见。推进“两区”建设，新增高标准农田11.9万亩，稳定粮食产量。大力实施农产品加工业提升行动，支持淮南（凤台）国家农业科技园区建设，支持温氏集团、正邦集团等农业企业形成规模，开工建设恒大高科农业生产基地，规上农产品加工产值增长6%以上。培育“三品一标”农产品20个。推进农业供给侧结构性改革，加快农业机械化和农机装备升级，合理调整“粮经饲”结构，积极培育生态农业、休闲农业、乡村旅游等新业态。加大农业新型经营主体培育，新增家庭农场150家、农民专业合作社100家。实施“互联网+”农产品出村工程，推广“电商企业+基地+合作社+农户”模式，新增农村电商经营主体300个，农村产品网络销售额增长30%以上。着力建设美丽乡村。持续推进厕所、垃圾、污水“三大革命”，同步启动“五清一改”清洁家园行动。完成5万户自然村农厕改造，开展246个建制村整治，建成30个省级中心村，加快乡镇政府驻地生活污水治理进程。完成“四好农村路”扩面延伸工程667公里、县乡公路大中修工程140公里。开工沉陷区安置房2600套，搬迁入住3100户。做好农田水利基本建设。完成农村安全饮水巩固提升任务。深化文明村镇创建。提升现代乡村治理能力。培育新型职业农民。支持少数民族和民族聚居地区优先发展。深化农村综合改革。推进农村集体产权制度改革，完成120个村“三变”改革，发展新型农村集体经济。开展农业社会化服务工作，扎实推进农村金融、供销社、集体林权和农业水价等改革。

（六）扎实推进新型城镇化，增强转型提速高质量发展的平衡性。坚持以人为核心的新型城镇化，促进区域协调发展，建设绿色城市、森林城市、海绵城市、智慧城市、人文城市、宜居城市。以“多规合一”的理念优化城市空间布局。把准淮南重点开发区域的功能定位，推进城市总体规划修编，在塑造城市特色和建筑风貌上系统谋划、有序推进。以整体开发的理念主攻山南。坚持定重点、提品质、塑形态、强特色、聚人气，加强建筑风貌、建设强度、建筑高度、城市色彩、第五立面、城市天际线等各方面管控。重点推进淮河大道两侧20平方公里核心区、国务院批准的5.15平方公里高新产业园区以及高铁南站周边商务区集中连片合围建设，打造全省一流的产城融合新区。开工山南新区综合医院、拓基商业综合体等12个项目，续建“双创”综合服务中心、金铂光电自动化设备生产等59个项目，建成淮南南站站前广场、新能源电池等9个项目，在商业综合体、社会办医、民办学校引进上实现新的突破。以“城市双修”的理念提升老城品质。坚持小投入、大管理，着力解决痛点、疏解堵点、破除难点。有序推进“两治三改”，改造老旧小区42个，开工城市棚户区（城中村）住房改造2433套。完善城市功能，继续实施老城区道路大中修改造工程，新改建各类管网100公里。开工建设花鸟市场，加快推进舜耕山风景区生态景观、老市政府大院景观及停车场改造。实施一批口袋绿地、景观绿道项目，让群众“出门见绿、起步闻香”。以统筹协调的理念构筑综合交通。畅通城市内循环，开工建设孤堆至毛集一级公路改建一期工程，加快推进学院路下穿铁路立交等项目。打通城市对外通道，加快推进6个普通国省干线“断头路”项目，实施国省干线公路大中修工程。推进3个综合客运站、3个综合客运枢纽建设，推动城市公交向农村延伸。以“绣花”的功夫提升城市治理水平。把创建全国文明城市融入到群众日常工作生活中，倡导“小手拉大手”，重点抓好占道经营、交通秩序等专项整治，营造“说文明话、做文明事、当文明人”的浓厚氛围。严格落实燃放烟花爆竹管理规定。强力治理违法建设、违法用地，大力实施城乡建设用地增减挂钩。建设智慧城管，提升城市治理科学化、精细化、智慧化水平。

（七）打好污染防治攻坚战，增强转型提速高质量发展的永续性。坚守阵地、巩固成果，大力实施“1＋7”污染防治行动计划，抓好环保督察反馈问题整改，帮助企业制定环境治理解决方案，满足群众对良好生态环境的新期待。打赢蓝天保卫战。聚焦“五控”，强化扬尘、散煤、“散乱污”企业、柴油货车等专项整治。用好大气监测平台，推进精细管控。严格执行重污染天气应急预案，抓好秸秆禁烧和综合利用工作，进一步改善空气质量。打好碧水保卫战。全面推行河（湖）长制，重点做好“五治”工作。加强饮用水水源地保护。实施城镇污水处理提质增效三年行动，稳步推进老城区雨污分流管网建设，协同推进城市排水防涝、黑臭水体整治及海绵城市建设，开展城镇和园区污水管网配套建设攻坚。加快城市建成区黑臭水体和入湖口治理，建成区黑臭水体消除比例达90%。严厉打击非法采砂行为。积极创建国家节水型城市。推进净土保卫战。围绕“五绿”，推深做实林长制改革。实施增绿补绿三年行动计划,完成成片造林1.08万亩。加大采石废弃地等生态脆弱区的森林生态系统修复。抓好固体废物和农业面源污染防治工作。强力推进开发区、风景名胜区等涉生态环保问题整治，开展园区厂居混杂问题、“大棚房”问题整治。坚决守住生态保护红线，让群众生活在天更蓝、山更绿、水更清的优美环境之中。

（八）提高保障和改善民生水平，增强转型提速高质量发展的普惠性。把握好政府过“紧日子”和让老百姓过“好日子”的关系，从小处着手、以小见大，高标准实施好省级民生工程，让发展更有温度、幸福更有质感。尽力办好群众身边事。建设街头游园10座。改造升级菜市场8个。新改建城市公厕24座。新增、延长公交线路3条。建成30条全民健身路径。开展虚假违法广告集中整治。完成四类重点对象存量农村危房改造。开展“送戏、送书、送电影”下乡活动。实施就业优先政策。坚持以创业带动就业，深入实施创业淮南行动计划，落实终身职业技能培训制度。落实求职和创业补贴、企业稳岗补贴政策。发放创业担保贷款2亿元。全年城镇新增就业4万人，农村劳动力转移就业3.7万人。办好优质公平教育。坚持教育优先发展战略，提高教育质量。推进县域义务教育优质均衡发展创建。持续实施第三期学前教育行动计划，新改扩建公办幼儿园10所。开展小区配套幼儿园移交清理工作。稳步实施普通高中新课程改革。积极推进社会办学和校企合作办学，加快发展职业教育。推动高等教育内涵式发展。提升居民消费能力。落实好个人所得税专项附加扣除政策，千方百计增加各项收入，有效激发居民消费潜力。从消费供给端发力，不断满足群众分层次多样性消费需求，让老百姓安心、舒心消费。完善社会保障体系。深入实施全民参保计划。稳步提高社会救助标准，继续推进城乡低保统筹和提标扩面，提升特困人员供养机构护理能力。加强欠薪源头治理，保障农民工工资支付。推进健康淮南建设。加快与安理大合作共建第一附属医院。推进市第五人民医院、市级老年公寓等项目建设，建成市传染病医院、市残疾人康复中心，加快乡镇卫生院建设，创建2个以上省级医养结合示范机构。加快体育强市建设，推动全民健身与全民健康深度融合。巩固省级食品药品安全城市创建成果。着力建设平安淮南。全力做好信访维稳工作。深化社会治安防控体系建设，深入开展扫黑除恶专项斗争，扎实推进“守护平安”行动，依法打击和惩治违法犯罪活动。积极开展“三社联动”试点。做好防灾减灾救灾工作。深入推进安全生产铸安行动，有效防范遏制安全生产事故。扎实推动军民融合深度发展。全面贯彻习近平强军思想，大力支持国防建设和军队改革，统筹做好国防动员、教育、优抚安置等工作，争创全国双拥模范城。充分发挥工会、共青团、妇联等群团组织的作用，做好地震、气象、档案、外事侨务、对台、妇女儿童、宗教和机关事务管理等工作，加快人民防空、广播电视、地方志、老龄、残疾人和红十字等各项事业发展。

**三、持续推进政府自身建设，坚决做到“两个维护”**

各位代表，时代是出卷人，我们是答卷人，人民是阅卷人。我们一定不忘初心、牢记使命，强化战略思维、辩证思维、创新思维、法治思维、底线思维，做好转型发展和从严治党两张报表，坚守五条工作底线，努力向全市人民交出合格答卷。

专业专心专注增本领。坚定不移提高政治站位，增强“四个意识”，坚定“四个自信”，坚决做到“两个维护”。带头弘扬社会主义核心价值观，压紧压实意识形态工作责任。加强理论武装，干什么学什么、缺什么补什么，切实增强八种本领，提升专业水平，提升抓经济、上项目水平，提升解决经济运行和项目建设难点堵点水平，真正经风雨、见世面、壮筋骨、长才干。

公开公平公正重法治。深化“七五”普法，完善政府工作人员学习宪法法律制度。深入推进依法行政，坚持按法规政策办事，按程序流程办事，按法律的主旨精神创新担当办事，加快建设法治政府。自觉接受党内监督、人大监督、政协民主监督，主动接受社会和舆论监督，认真办理人大代表议案建议和政协委员提案。坚持科学、民主、依法决策，全面推进政务公开，让权力在阳光下运行。

认真较真动真抓落实。一分部署,九分落实。坚定实干兴淮，完善工作压力传导和责任分解体系，创造性落实中央和省市委决策部署，以真考核、严奖惩提升政府执行力和公信力，使工作有兴奋点、落实有发力点、经济有增长点、发展有新亮点。全面提振精气神，健全激励约束、容错纠错机制，旗帜鲜明为担当者担当、为负责者负责。坚守大情怀，始终把全市人民对美好生活的向往作为奋斗目标，以踏石留印、抓铁有痕的劲头狠抓任务落实。

实干苦干巧干提效能。为人民干事是天职，不干是失职。发挥“头雁效应”，争当奋斗者、实干家。继续开展“全面学合肥，对标提效能”活动，让见贤思齐、奋勇争先成为共识，让马上就办、办就办好蔚然成风。切实改进文风会风，统筹规范督查检查考核，让基层有更多的时间抓落实、干实事。大兴调查研究之风，“身入”“心至”基层群众，吃透上情、掌握市情、熟悉下情，推动干部在一线干事、问题在现场解决、工作在全国全省大格局中谋划。

清正清廉清明守底线。认真履行全面从严治党主体责任，落实“一岗双责”。持之以恒执行中央八项规定精神及实施细则，坚定不移纠“四风”、树新风。深化廉政风险防控，加强对财政资金使用、公共资源交易、国有资产管理等重点领域的监管。支持纪检监察机关依法依规履行职责，强化行政层级监督，突出巡视巡察和审计结果运用，真正让制度“长牙”、纪律“带电”。

各位代表！蓝图目标不是等得来、喊得来的，而是拼出来、干出来的。让我们更加紧密地团结在以习近平同志为核心的党中央周围，在市委的坚强领导下，继续发扬敢闯敢拼不服输的精神，保持定力，真抓实干，为决胜全面建成小康社会，加快建设现代化五大发展美好淮南而努力奋斗！
